# Nomogram construction and validation of axial deviation in patients with tibial defects treated with the Ilizarov bone transport technique

**DOI:** 10.1186/s12891-024-07603-x

**Published:** 2024-06-19

**Authors:** Jinghong Yang, Zi Wang, Lujun Jiang, Lian Tang, Zhong Li, Yanshi Liu

**Affiliations:** 1 Department of Orthopedics, Affiliated Hospital of Southwest Medical University, Lu Zhou, 646000 People’s Republic of China; 2 Sichuan Provincial Laboratory of Orthopaedic Engineering, Lu Zhou, 646000 People’s Republic of China; 3 Stem Cell Immunity and Regeneration Key Laboratory of Luzhou, Lu Zhou, 646000 People’s Republic of China

**Keywords:** Bone transport, External fixation, Ilizarov technique, Nomogram

## Abstract

**Introduction:**

The Ilizarov bone transport technique is widely recognised as an effective method for treating large segment bone defects in clinical practice. However, axial deviation is a common complication in the treatment of tibial large segment bone defects, which can have a serious impact on the clinical efficacy of bone transport. Our study aims to construct and validate a nomogram for predicting axial deviation of tibial bone transport.

**Method:**

This study retrospectively collected data from 363 patients who underwent the tibial Ilizarov technique for bone transport. Univariate and multivariate logistic regression analyses were performed to determine the independent risk factors for axial deviation, which were later used to construct a nomogram. The nomogram was evaluated using the decision curve analysis (DCA), the calibration curve, and the area under the receiver operating characteristic curve (AUC).

**Results:**

Of the 363 patients who underwent Ilizarov tibial bone transport, 31.7% (115/363) experienced axial deviation. Multivariate logistic regression analysis showed that gender, height, defect site, and external fixation index were important risk factors for axial deviation. The AUC value of the nomogram model was 0.705. The calibration curve and the decision curve analysis showed a good consistency between the actual axial deviation and the predicted probability.

**Conclusion:**

The model assigns a quantitative risk score to each variable, which can be used to predict the risk of axial deviation during tibial bone transport.

## Introduction

Axial deviation is a frequently observed clinical complication that occurs when applying Ilizarov bone transport technology [1–3]. It manifests as a misalignment of the lower limb force lines and abnormal positioning of the transported bone segment. This displacement may occur during the transport process when the bone segment reaches the mating end. Multiple reasons can contribute to axial deviation, including excessive soft tissue extension or inadequate stabilization of the bone segment [4–6]. Axial deviation has several adverse consequences, including non-union, delayed union, infection, and even amputation [7, 8]. Therefore, it is crucial to assess the risk of axial deviation as soon as possible and to proactively prevent and manage axial deviation during Ilizarov bone transport surgery.

Some studies have shown that axial deviation significantly affects the outcome of bone transport in tibial bone defects [2, 3, 9–11]. Axial deviation not only exerts a detrimental effect on the adjacent joint but also elevates the risk of delayed healing, non-union, and refracture at the buttress end. Consequently, preventing axial deviation is of paramount importance in the management of large tibial bone defects.

A retrospective analysis was conducted on patients with tibia defects admitted to the Affiliated Hospital of Southwest Medical University between 2010 and 2021 who underwent Ilizalov bone transport technology. The study aimed to identify relevant risk factors for axial deviation and to conduct a nomogram prediction model to assist orthopedic clinicians in preventing its occurrence.

## Methods and materials

### Patients

This retrospective study was conducted by the Declaration of Helsinki and received approval from the Institutional Review Board of the Affiliated Hospital of Southwest Medical University. In our retrospective study, spanning from 2010 to 2021, a total of 363 patients presenting with tibial bone defects were effectively treated utilizing the Ilizarov bone transport technique. The inclusion criteria for this study were as follows: (1) age between 14 and 70 years; (2) tibial bone defect with a length of ≥ 3 cm; and (3) At least two years of follow-up after frame removal. The exclusion criteria were as follows: (1) systemic diseases, including liver or kidney insufficiency or diseases related to bone metabolism; (2) nerve or blood vessel injury or disease in the affected limb; and (3) poor compliance or loss to follow-up; (4) Patient lacks complete imaging data.

### Surgical technique

Firstly, a thorough examination was conducted to assess surgical contraindications, and the wound was meticulously debrided under general or epidural anesthesia. Before bone transport, all hardware was removed, necrotic and infected bone and soft tissues were thoroughly debrided, and antibiotic-impregnated cement spacers were implanted as necessary to enhance stability and promote healing. If infection was present, surface secretions and deep tissue scrapings were retained for bacterial culture and drug susceptibility testing to guide subsequent anti-infective therapy. When dealing with infected bone segments, it is important to ensure complete excision until the bone cortex stops oozing blood and the medullary cavity is reopened, which is commonly referred to as the “Paprika sign” [12, 13]. Small soft tissue defects are reconstructed using local tissue flaps or direct tension-free sutures, while flap transfers or free skin grafts are used to cover larger wounds.

Bone transport can be initiated when clinical signs and laboratory indicators indicate that the infectious process has ended. Typically, the waiting period for infection control is about two weeks following debridement. For patients who have exceeded this two-week mark, we assess the situation individually and often proceed with an additional osteotomy. Preoperative anteroposterior and lateral radiographs are used to assess the size of the defect and plan the construction of the external fixation. The type of external fixation is determined by the location of the bone and soft tissue defects, as well as the surgeon’s experience and the patient’s preference. The surgeon carefully selects the appropriate components and constructs the external fixator according to the defect size, ensuring accurate fit and placement. With regards to the Orthofix Fixation, we employ three 6.5 mm screws for the fixation of each block. Conversely, for the Ring Fixation, we utilize at least two tensioned wires for each block (exerting a force of 1200 N) along with one screw for fixation. Osteotomies are performed using the minimally invasive Gigli-saw technique, with special attention given to preserving as much periosteum as possible. All operations are performed by the same surgical team.

### Data collection

Demographic data included age, sex, weight, and height (BMI = weight (kg)/height (m2)), Defective part (proximal, middle, and distal), Defect size (Bone defect length, Soft-tissue defect length and width), Mechanisms of injury, Underlying comorbidities, Type of external fixation (circular (TrueLok Ring Fixation System, Orthofix, Verona, Italy) or monolateral (Limb Reconstruction System, LRS, Orthofix, Verona, Italy)).

Postoperative data included docking time, regenerate consolidation time, external fixation time, external fixation index (EFI), and axial deviation. The external fixation time (EFT) and the external fixation index (EFI) were defined as follows: EFT is the duration in days from the external fixation placement to its removal, while EFI is the ratio of EFT in days to the size of the bone defect. According to Paley’s classification criteria for complications in Ilizarov bone transport, an axial deviation is considered present if the force line at the docking end is > 5° [14].

### Statistical analysis

Data analysis was carried out using SPSS v26.0 software for Windows (IBM Corp., Armonk, NY, USA), and the nomogram was constructed and validated using R software (version 3.6.5, R Foundation for Statistical Computing, Vienna, Austria). The study’s sample size was determined using logistic regression analysis. In a logistic regression analysis, the sample size should be at least ten times the number of covariates to ensure reliable and accurate results [15]. In our study, there are 17 covariates, thus the minimum required sample size should be greater than 170. The study aimed to identify possible risk factors for axial deviation. Univariable logistic regression analysis was performed to determine these factors. Statistically significant variables (*p* < 0.05) from the univariable analysis were included in the multivariable logistic regression analysis using a stepwise procedure to identify independent risk factors. The prediction accuracy of the nomogram was assessed using the receiver operating characteristic (ROC) curve areas, and a calibration curve was plotted to evaluate the calibration ability of the nomogram. A decision curve analysis (DCA) was applied to evaluate the net benefit. The predictive model was built based on a training cohort. The accuracy of this nomogram was then validated in a validation cohort obtained by random sampling from the total population using the same method described above.

## Result

### Patients’ characteristics

A total of 355 patients with tibial bone defects admitted to our hospital between January 2010 and December 2021 underwent Ilizarov bone transport. The training cohort comprised 290 patients, of whom 91 (31.4%) exhibited axial deviation. All data of patients, including demographic, preoperative, and postoperative data in all patients, are given in Table 1.

**Table 1:** Demographics and clinical characteristics of patients

Variables	total	Occurrence	Control	*p*
Gender				0.763
male	303	95(31.4)	208(68.6)	
female	60	20(33.3)	40(66.7)	
Age				< 0.001
>=40	177	40(22.6)	137(77.4)	
< 40	186	75(40.3)	111(59.7)	
Height				0.008
>=1.65	249	68(27.3)	181(72.7)	
< 1.65	114	47(41.2)	67(58.8)	
Weight				0.773
>=70	165	51(30.9)	114(69.1)	
< 70	198	64(32.3)	134(67.7)	
BMI				0.637
<=25	224	73(32.6)	151(67.4)	
> 25	139	42(30.2)	97(69.8)	
Mechanisms of injury				0.336
car accident injury	92	23(0.25)	69(0.75)	
falling injury	45	13(28.9)	32(71.2)	
Sharps Injury	7	3(42.9)	4(57.1)	
bruise	219	76(36.1)	143(63.9)	
Underlying comorbidities				0.008
no	316	91(28.8)	225(71.2)	
hypertensive	14	5(35.7)	6(64.3)	
diabetes	33	16(48.5)	17(51.5)	
Defective part				0.003
proximal	42	8(19.0)	34(81.0)	
middle	168	68(40.0)	100(60.0)	
remote	153	39(25.5)	114(74.5)	
Bone defect length				< 0.001
<=5 cm	125	21(16.8)	104(83.2)	
> 5 cm,<10 cm	215	81(37.7)	134(62.3)	
>=10 cm	23	13(56.5)	10(43.5)	
Soft-tissue defect				0.828
yes	43	13(30.2)	30(69.8)	
no	320	102(31.9)	218(68.1)	
Soft-tissue defect length				0.997
>=5 cm	41	13(31.7)	28(68.3)	
< 5 cm	322	102(31.7)	220(68.3)	
Soft-tissue defect width				0.633
>=5 cm	28	10(35.7)	18(64.3)	
< 5 cm	335	105(31.3)	230(68.7)	
Type of external fixator				0.199
Orthofix Fixator	327	107(32.7)	220(67.3)	
Ring External Fixatior	36	8(22.2)	28(77.8)	
Meeting Time				0.001
<=60	46	5(10.9)	41(89.1)	
> 60,<90	206	63(30.6)	143(69.4)	
>=90	111	47(42.3)	64(57.7)	
Mineralisation Time				< 0.001
<=200	42	6(14.3)	36(85.7)	
> 200.<300	273	81(29.7)	192(70.3)	
>=300	48	28(58.3)	20(41.7)	
Wearing time				0.001
<=300	44	5(11.4)	39(88.6)	
> 300,<=400	206	61(29.6)	145(70.4)	
> 400,<=500	92	38(41.3)	54(58.7)	
500	21	11(52.4)	10(47.6)	
EFI				< 0.001
<=40	58	34(58.6)	24(41.4)	
> 40,<=50	17	4(23.5)	13(76.5)	
> 50,<=60	49	15(30.6)	34(69.4)	
> 60,<=70	166	48(28.9)	118(71.1)	
> 70	73	14(19.2)	59(90.8)	

abbreviation: EFI: external fixation index

### Risk factors associated with axial deviation

Table 2 presents the risk factors associated with axial deviation, which were significantly elevated in univariate analysis. Table 2 also demonstrates the independent risk factors linked to axial deviation in patients with tibial bone defects undergoing Ilizarov bone transport. These factors were identified through multivariable regression analysis after adjusting for confounding variables. Notably, Age (OR = 2.549; *P* = 0.009), Height (OR = 0.461; *P* = 0.027), Defective Part (*P* = 0.019), and EFI (*P* = 0.003) emerged as significant predictors.

**Table 2:** The results of Univariate and Multivariable logistics regression analysis

Variables	Univariate		Multivariable	
	OR(95%CI)	*P*	OR(95%CI)	*P*
Gender(male/female)	1.066(0.533–2.130)	0.857		
Age	2.618(1.558–4.399)	< 0.001	2.549(1.257–5.167)	0.009
Height	0.537(0.316–0.912)	0.021	0.461(0.233–0.915)	0.027
Weight	0.938(0.571–1.543)	0.802		
BMI	1.041(0.628–1.726)	0.877		
Mechanisms of injury		0.390		
car accident injury	1			
falling injury	0.576(0.304–1.091)	0.576		
Sharps Injury	0.762(0.354–1.640)	0.762		
bruise	0.935(0.167–5.251)	0.935		
Underlying comorbidities		0.004		
no	1			
hypertensive	0.330(0.145–0.748)	0.008		
diabetes	1.200(0.301–4.782)	0.796		
defective part		0.004		0.019
proximal	1		1	
middle	0.653(0.229–1.861)	0.425	1.724(0.507–5.865)	0.383
distal	2.166(1.270–3.694)	0.005	2.570(1.332–4.960)	0.005
Bone defect length		< 0.001		
<=5 cm	1			
> 5 cm,<10 cm	0.111(0.035–0.352)	< 0.001		
>=10 cm	0.354(0.123–1.017)	0.054		
Soft-tissue defect	1,770(0.775–4.041)	0.175		
Soft-tissue defect length	0.614(0.267–1.410)	0.250		
Soft-tissue defect width	0.892(0.356–2.233)	0.807		
Type of external fixator	0.568(0.222–1.452)	0.568		
Meeting Time		0.003		
<=60	1			
> 60,<90	0.193(0.069–0.536)	0.002		
>=90	0.565(0.331–0.963)	0.036		
Mineralisation Time		< 0.001		
<=200	1			
> 200.<300	0.133(0.046–0.387)	< 0.001		
>=300	0.288(0.145–0.574)	< 0.001		
Wearing time		0.003		
<=300	1			
> 300,<=400	0.131(0.034–0.498)	0.003		
> 400,<=500	0.349(0.127–0.963)	0.042		
> 500	0.652(0.227–1.868)	0.426		
EFI		< 0.001		0.003
<=40	1		1	
> 40,<=50	6.072(2.517–14.647)	< 0.001	11.160(2.636–47.247)	0.001
> 50,<=60	0.388(0.045–3.334)	0.389	0.441(0.040–4.898)	0.505
> 60,<=70	1.915(0.758–4.839)	0.169	1.602(0.432–5.938)	0.481
> 70	1.792(0.842–3.814)	0.13	1.619(0.589–4.453)	0.350

abbreviation: EFI: external fixation index

### Nomogram construction and validation

**Fig. 1:**
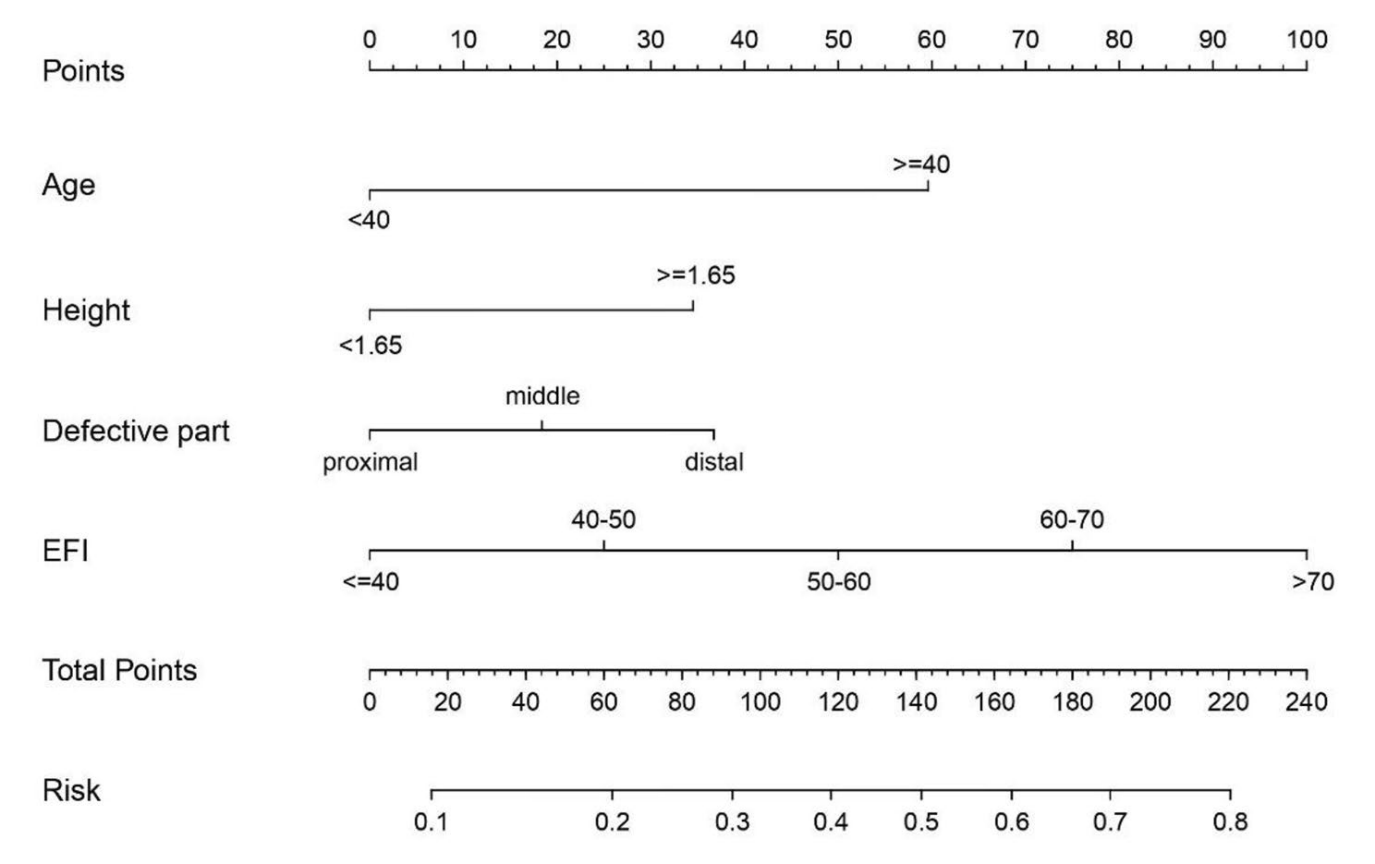
Nomogram for predicting axial deviation in patients with tibial bone defect receiving Ilizarov bone transport

**Fig. 2:**
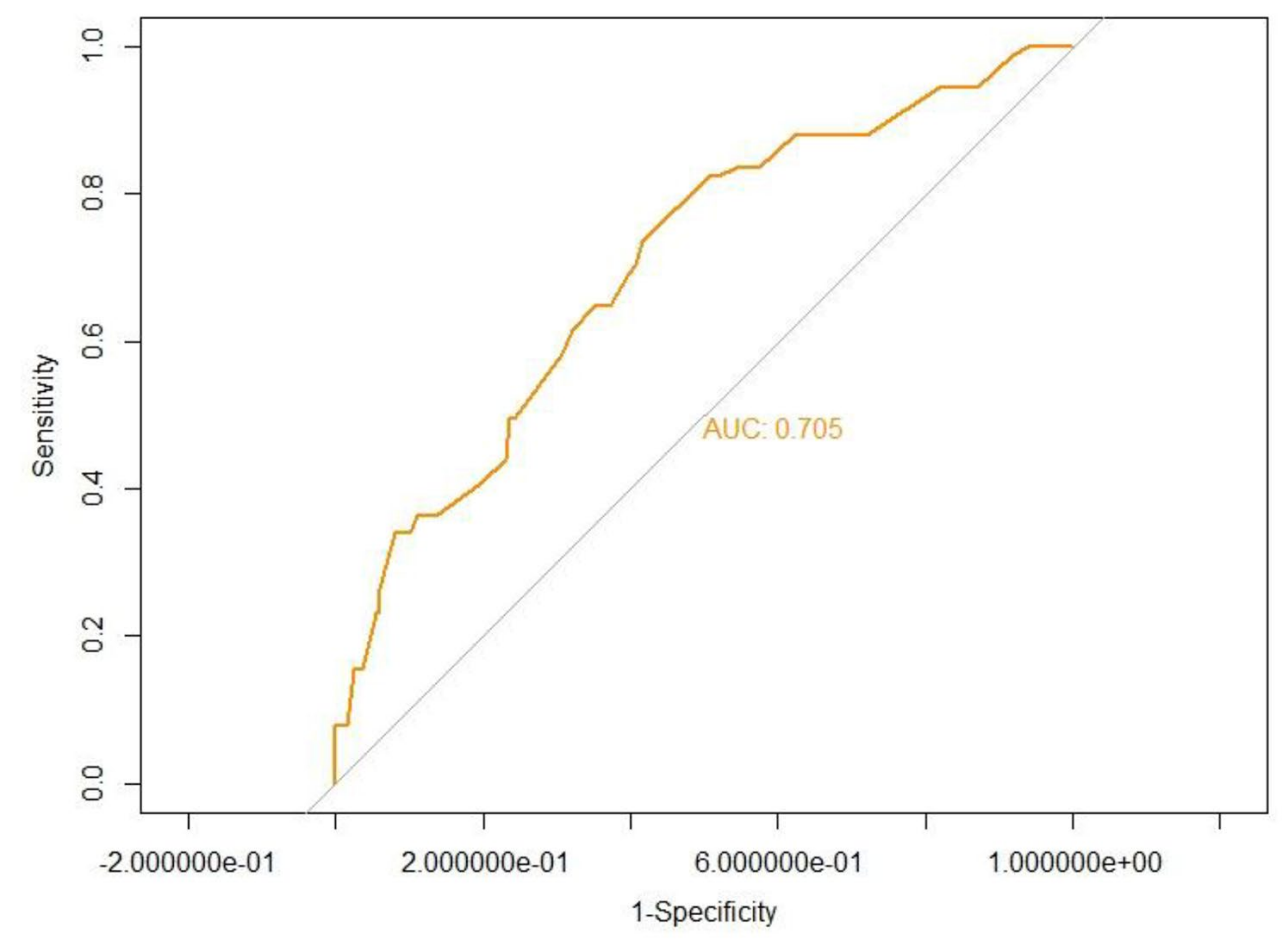
The ROC analysis for the predictive model

**Fig. 3:**
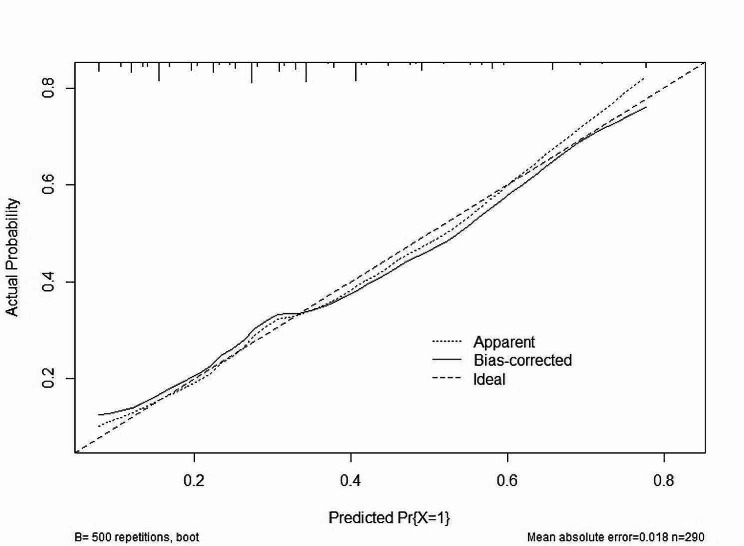
The calibration curve indicated good consistency between the actual diagnosed axial deviation and the predicted probability

**Fig. 4:**
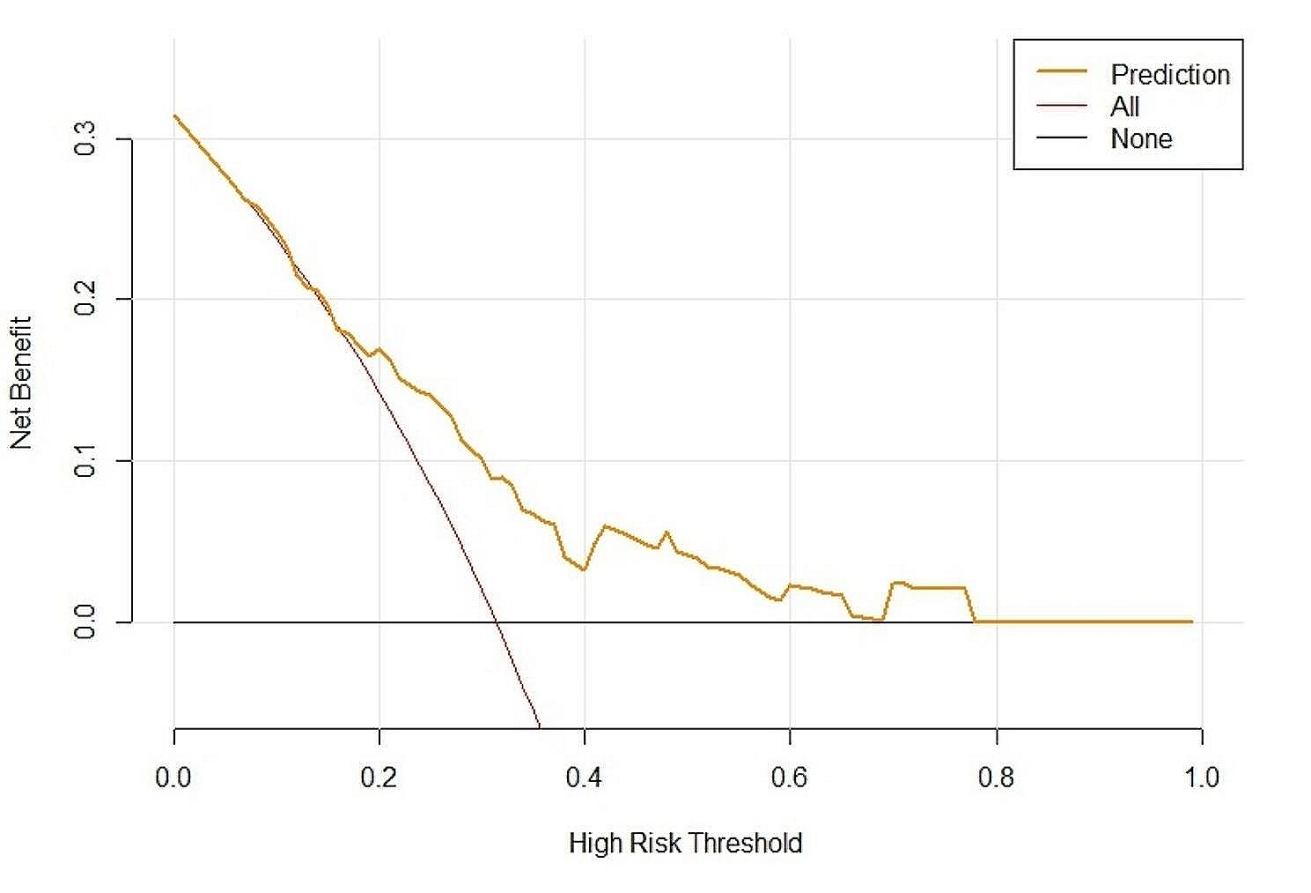
Decision curve analysis (DCA) of the nomogram

A nomogram of quantitatively predicted axial deviation was constructed using independent risk factors identified through multivariate analysis (Fig. 1). The ROC curve was constructed to demonstrate the high discriminatory power of the model, with an AUC value of 0.704 (Fig. 2). Calibration curves and DCA curves demonstrate good agreement between actual axial deviation and predicted probabilities, indicating that the model is both accurate and reliable (Figs. 3 and 4).

## Discussion

As clinical prediction models have been widely used in tumor prognosis, their application in general disease prediction is becoming increasingly prevalent. In orthopedics, clinical prediction models have been extensively utilized to predict surgical outcomes, disease prognosis, and post-operative complications [16–20]. However, no prognostic nomogram has been developed to predict axial deviation following Ilizarov tibial bone transport. Therefore, we have innovatively developed a nomogram to predict the risk of axial deviation following Ilizarov tibial bone transport by analyzing various procedure-related factors. Ilizarov tibial bone transport is a widely utilized surgical approach for treating large tibial bone defects. During bone transport, there is often axial deviation due to the tibia’s physiological curvature and the requirement for mechanical linear motion during bone transport, which can lead to a shift in the line of force on the affected side. This can lead to a delay in buttress end healing and an increased risk of re-fracture [7].

The formation of new bone during bone transport is not only influenced by the biomechanical environment but also by the osteogenic potential of the osteotomy site. Therefore, in clinical practice, the metaphysis is often the preferred site for osteotomy due to its abundant cancellous bone and rich blood supply, which facilitate the growth of new bone [21, 22]. However, Aarnes et al. [23] observed that the osteocarrying segments of proximal tibial osteotomies may exhibit varying degrees of offset deformity. This could be attributed to the positioning of the gastrocnemius muscle group primarily on the posterior-lateral side, which exerts strain on the truncated end of the osteotomy. Multiple studies have documented complications related to axial deviation following Ilizarov tibial bone transport. Feng et al. [1]conducted a retrospective analysis of 103 patients undergoing tibial bone removal and identified axial deviation in 19 patients. Their findings revealed a significant correlation between the length of the bone defect and the duration of external fixation with axial deviation. In a separate retrospective study, Feng et al. [24]examined 199 patients with tibial bone defects and observed axial deviation in 86 cases. Notably, there was a significant correlation between axial deviation and bone defects located in the middle third of the tibia, as well as the length of the bone defects and EFI. This alignment with our findings underscores a robust correlation between EFI, defect site, and other variables, and the incidence of axial excursion. In a retrospective analysis of 282 consecutive cases over 10 years, Liu et al. [7]reported 82 cases of axial deviation in a retrospective analysis of 282 consecutive cases over a 10-year period, while Gamal Ahmed Hosny [7] identified 21 patients with re-fracture among 812 patients treated with the Ilizarov bone transport technique for infected tibial malunion, of whom 4 had axial deviation. However, the existing studies primarily emphasized the reporting and treatment of axial deviation, while failing to conduct a comprehensive analysis of the underlying causes and risk factors of axial deviation. Furthermore, they did not establish predictive models for the prediction of axial deviation.

In our study, the univariate and multivariate logistic regression analysis showed that gender, height, defect site, and external fixation index (EFI) were important risk factors for axial deviation. Multiple studies have examined the association between gender, defect site, and EFI with axial deviation, reporting various findings [7, 24, 25]. Firstly, the gender factor potentially exerts a certain degree of influence on axial deviation during bone transport procedures. Specifically, anatomical variations between males and females, such as disparities in bone structure, density, and volume, can contribute to disparities in bone stability and fixation during these surgical interventions. Furthermore, gender-specific physiological and endocrine differences may influence the healing and regenerative capacities of bone, potentially leading to distinct outcomes in men and women regarding the occurrence of axial deviation. Secondly, height is intricately linked with axial deviation. Typically, an individual’s height correlates with bone length, proportionality, bone density, and overall bone strength. Varying heights can result in distinct bone structures and biomechanical properties, which, in turn, may influence the stability of bones when subjected to external forces during surgical manipulation. Furthermore, the specific site of the bone defect and the EFI serve as pivotal factors in influencing axial deviation. Varying defect locations can exert distinct impacts on bone stability and force distribution patterns. Notably, defects situated in load-bearing regions, particularly proximate to distal places or joints, may heighten the likelihood of axial deviation occurrence. The external fixation index serves as a reliable metric for gauging the stability and functionality of the external fixation system. A superior external fixation index is indicative of a more robust fixation system, capable of resisting external stresses and maintaining bony alignment. Therefore, axial deviation arises as a multifaceted outcome, stemming from a confluence of these factors.

However, further research is needed to clarify the exact relationship between these factors and axial deviation, especially in different patient populations and clinical settings. Gigli saw osteotomy is used more often in the surgeries performed in our center, compared to the De Bastiani technique, since there is minimal periosteal disruption and limited concern of thermal necrosis in Gigli saw osteotomy. Published studies have demonstrated that fresh bone healing tissue possesses the capacity to differentiate and survive within the transport gap, but it is also vulnerable to mechanical stimulation from external forces, which can influence its growth direction [26, 27]. Numerous studies have demonstrated the advantages of unilateral external fixation frames, including simplicity of installation and high patient acceptance, which are associated with poor stability of the entire frame. Furthermore, the two-dimensional spatial structure can result in uneven distribution of force lines, ultimately leading to deformation [28, 29]. However, Some research has further demonstrated the absence of significant differences in the fixation strength between orthofix external fixation and ring external fixator, indicating that both techniques offer robust stabilization for distraction osteogenesis [30, 31]. Multiple studies conducted by Aihematijiang Yusufu et al. [32, 33] have consistently revealed that orthofix external fixation exhibits superior efficacy in the treatment of bone defects when compared to Ilizarov ring external fixation. Notably, patients in the orthofix group exhibited greater satisfaction with their quality of life and post-surgical outcomes, while experiencing relatively less negative psychological impact. Nevertheless, previous studies have not deeply explored the effect of the two types of external fixation on axial deviation, and our study also did not observe a statistically significant difference in the risk of axial deviation between the ring external fixation frame and the orthofix external fixation frame.

In our study, we constructed a nomogram to accurately predict the presence of axial deviation in Ilizarov tibial bone transport. Initially, we selected 17 potential risk factors based on previously published literature. Subsequent univariate and multivariate logistic regression analyses identified 4 independent risk factors that can be easily applied in clinical practice.

In conclusion, our nomogram offers an accurate prediction of axial deviation following Ilizarov tibial bone removal. We employed ROC curves to assess the accuracy of the model, and the high value of AUC indicated the nomogram provided a more precise risk assessment. Furthermore, calibration curves and DCA curves demonstrate good agreement between actual axial deviation and predicted probabilities, indicating that the model is both accurate and reliable. By using this model, orthopedic clinicians can individually assess the risk of axial deviation, enabling them to take proactive measures to decrease the likelihood of axial deviation and prevent the occurrence of lower limb force line misalignment and malformed healing. However, this study possesses certain limitations. Firstly, it is a single-centre retrospective study with a relatively small sample size. Secondly, only four independent risk factors were screened in this analysis, but axial deviation after Ilizarov tibial transport may be affected by a variety of factors, such as muscle injury, postoperative care, smoking, osteoporosis, and other factors. Multiple studies have highlighted the significant impact of smoking on osteogenesis, while osteoporosis and bone loss are also potential factors contributing to suboptimal screw fixation. Unfortunately, due to the retrospective nature of the study, we did not include these influences in our study. Thirdly, although we applied multiple ways to validate the accuracy of the predictive model, before using the predictive model in clinical practice, he should have tested it externally in another multicenter, large sample size model.

## Data Availability

The datasets used and/or analyzed during the current study are available from the corresponding author upon reasonable request.
